# Maxillary sinus aspergillosis: a case report of the timely failure to treatment

**Published:** 2019-08

**Authors:** Asma Beyki, Mahmud Zardast, Zahra Nasrollahi

**Affiliations:** 1 Department of Medicine, Birjand University of Medical Sciences, Birjand, Iran; 2 Department of Pathology, School of Medicine, Birjand University of Medical Sciences, Birjand, Iran; 3 Faculty of Paramedicine, Qom University of Medical Sciences, Qom, Iran

**Keywords:** Aspergillosis, Maxillary sinusitis, Fungal ball

## Abstract

Invasive aspergillosis of the paranasal sinuses is a rare and often misdiagnosed disease. This study reported a case of maxillary aspergillosis with a complete headache and eye pain after tooth extraction with a large abscess in the relative jaw. Tenderness in the right temporal, lower jaw numbness and right eye proptosis was found. Histopathological examination was the suggestion of maxillary sinusitis with a fungal ball of aspergillus.

## INTRODUCTION

Sinusitis aspergillosis currently constitutes the most common cause of opportunistic fungus infectious in immunocompromised patients and classified as invasive or noninvasive. Furthermore, infection of the maxillary sinus may be complicated by direct invasion into the palate with necrosis and perforation into the oral cavity or perforation of the nasal septum. Worldwide, *Aspergillosis flavus* and *A. fumigatus* are the most common species which often involve the maxillary sinus (1). The clinical manifestation of aspergillosis varies, depending on the host immune status and the presence or absence of tissue damage.

Another presentation that may be encountered by the oral healthcare provider is aspergillosis after tooth extraction or endodontic treatment, especially in the maxillary posterior segments. Presumably, tissue damage predisposes the sinus to infection, resulting localized pain and tenderness accompanied by nasal discharge.

Fungal rhinosinusitis is classified into an invasive and a non-invasive form. The non-invasive form are allergic sinusitis and aspergilloma. This infection leads to the destruction of the sinus mucosa, bone atrophy, eosinophilia (2, 3) and elevated allergic mucin with Charcot-Leyden crystals (4).

Invasive aspergillosis can be either limited [chronic or indolent] or fulminant [acute] (3, 5). However, in both states, hyphae can invade the sinus mucosa, bone, orbital tissue, even along the skull base and larger vessels leading to cavernous sinus thrombosis and a variety of central nervous system manifestations within a few days (6, 7). Intracranial and intraorbital extensions decrease the survival rate and increase surgical morbidity. Clinicoradiological findings can be misleading as the lesions are locally destructive and mimic a neoplasm. A biopsy is necessary to establish the diagnosis. Hyphae are typical and specific for each fungus. Mucor presents large, broad nonseptate hyphae with right-angle branching, and aspergillus shows septate hyphae that branch at 45° angles. The histology should be specific; whether there is mucosal involvement [invasive] or the mucosa is intact [non-invasive disease]. Fungal cultures on sabouraud’s dextrose agar are needed to confirm the diagnosis (8, 9).

Treatment depends on the clinical presentation of aspergillosis. For immunocompetent patients with a noninvasive aspergilloma, surgical debridement may be necessary. Patients who have allergic fungal sinusitis treated with debridement and corticosteroid drugs. For localized invasive aspergillosis in the immunocompetent host, debridement followed by antifungal medication is indicated. Although, systemic amphotericin B deoxycholate therapy was considered appropriate in the past, studies showed that voriconazole, a triazole antifungal agent, is more effective for treatment. Among most patients with invasive aspergillosis, 71% of those who treated with voriconazole were alive after 12 weeks of therapy, compared with 58% survival in the group who received standard amphotericin B or caspofungin debridement of necrotic tissue combined with systemic antifungal therapy.

Although, much progress has occurred in the last 20 years, 50% of those with invasive aspergillus type are to be diagnosed after death (10). Give the similarity between fungal sinusitis symptoms and chronic bacterial sinusitis as well as the increased incidence of aspergillosis, early diagnosis and treatment are important (11).

This study describes a case of maxillary aspergillosis in an individual after tooth extraction with a large abscess in the relative jaw. Histopathological examination in the cases of maxillary sinusitis due to aspergilloma can be the best strategies to improve early diagnostic effectively and efficiently without leaving any inability.

## CASE

The patient was a 58-year-old man admitted to Vali Asr hospital of Birjand, Khorasan province of Iran, who complained a complete headache and eye pain beginning 10 hours after the extraction of the third right molar, while there was no hemorrhage and blood spot on the wound dressing. He had a history of type 2 diabetes Mellitus without nephropathy. He mentioned a drug history of prednisone, losartan, metformin and glibenclamide pills. After the examination, swelling and inflammation in the right side of the face, right eye proptosis, swelling and severe purulent inflammation around the left eye leading to its closure, tenderness in the left temporal and numbness of the lower jaw were found. The axillary temperature was 37°C and blood pressure was 150/96 mmHg.

**Fig. 1:**
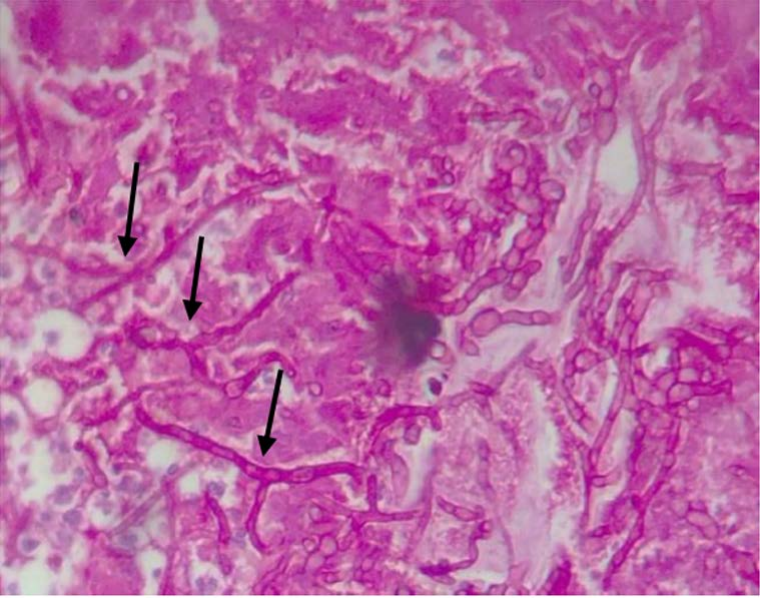
Histological examination (hematoxylin and eosin stain) shows abundant septate fungal hyphae with dichotomous branching suggestive of the Aspergillus

In the laboratory study, high levels of white blood cells (19310.1mm3), Erythrocyte Sedimentation Rate (51), platelets (628000.1 mm3) as well as low levels of MCV (77.7 FL) and MCH (25.9 Pg) were found. An otolaryngologist was consulted and debridement by surgery was arranged. After surgery, samples were sent to the pathology laboratory. Based on the data and the pathology report, small pieces of tissue with collective dimensions of 1×0.6×0.3 cm in cream and brown colors and consistency of soft fixed were sent. In microscopic examination of the prepared smears, microtic tissue with fungal ingrowth compatible with aspergillus was seen (Fig. 1); Mucor was not seen.

According to the patient’s history and clinical study, the treatment was wrapped. The diagnosis was facial paresis and aspergillosis. He was treated with Oint Vitamin A every 4 hours in the right eye, artificial tear drop every 4 hours in the right eye, Amp Ceftazidime 1 gr IV every 12 hours, Amp Vancomycin 1 gr IV every 12 hours, Amp Clindamycin 6 mg IV every 8 hours, Amp Dexamethasone 4 mg IV every 12 hours and Amp Amphotericin B-Lyophilized 50 mg IV, daily. After a week, his headache was relatively improved and he was discharged. After two months, aspergillosis was treated, but complications of vascular and neurological infection involvement including left blindness and right facial paralysis were continuing in the follow-up.

## DISCUSSION

Fungal sinusitis constitutes 6–9% of all rhinosinusitis (12). *A. fumigatus* (80–90%) is the most common species and maxillary sinus is the most affected through the world. *A. flavus* (5–10%), *A. niger* (1–5%) and *A. terreus* (1%) are less common (13). Fungal rhinosinusitis is classified into invasive and non-invasive forms. The non-invasive forms are allergic sinusitis, and invasive aspergillosis can be either limited (chronic) or fulminant (acute) (3, 5). The most common symptoms of fungal sinusitis including headache, proptosis, rhinorrhea and ophthalmoplegia (5, 10, 13, 14) were observed in our patient. Neutropenia and glucocorticoid are the most common predisposing factors (10). Fungal rhinosinusitis is frequently found in patients with uncontrolled diabetes mellitus who are in an immunocompromised state such as using glucocorticoids (4, 7, 15).

Invasive fungal sinusitis must be considered in immunocompromised or diabetic patients who present with acute sinusitis, inflammation of nasal septal mucosa, unexplained fever or cough. Different forms of aspergillosis appearance showed in literature. Choi et al. reported a case in whom aspergillosis of the paranasal sinuses presented like an optic neuritis (16).

Diabetes mellitus is a group of metabolic diseases characterized by high blood glucose levels (hyperglycemia) and the inability to produce and/or use insulin (17). As indicated by the data for general surgery procedures, if the fasting blood glucose level is below 206 mg/100 mL, increased risk is not predicted. However, if fasting blood glucose level is between 207 and 229 mg/100 mL, the risk is predicted to be increased by 20% if surgical procedures performed. Additionally, if fasting blood glucose level rises above 230 mg/100 mL, an 80% increase risk of infection postoperatively reported (17). Although, these studies predict risk based on non-oral surgical procedures, dentists should be aware of the level of glycemic control among patients undergoing complex oral surgical procedures because of the predicted increased risk of infection. Judicious monitoring and an appropriate use of antibiotics should be considered (18). In our patient, thickening of the right maxillary sinus was observed and after consulting with an otolaryngologist, debridement by surgery was arranged. Histopathological examination showed microtic tissue holding fungal ingrowth with septate hyphae and branch at 45° angles compatible with aspergillus.

The primary treatment for aspergillosis includes surgical debridement and antifungal therapy. For those that total removal cannot be achieved, intensive therapy with antifungal agents, in particular voriconazole as first line therapy or in case of chronic renal failure as second line therapy liposomal amphotericin B must be started immediately (19). The outcome, however, is poor among most of them. It was recently showed the combination of voriconazole and caspofungin or amphotericin B and itraconazole for skull base aspergillosis might represent a step forward in the treatment of invasive aspergillosis (20).

It is important to distinguish the invasive disease from the non-invasive because of different treatment and prognosis. Since fungal infections occur infrequently, they might pose a diagnostic and therapeutic dilemma for those who are not familiar with its clinical presentation. Early diagnosis is vital in these infections; delayed initiation of treatment can be life threatening due to the propensity of the fungi to invade adjacent blood vessels whereby the connective tissue produces thrombosis and ultimately necrosis of the hard and soft tissues (21). Moreover, biopsy of sinus must be planned and performed rapidly. CT and MRI scans are useful diagnostic tools for detecting calcified aspergillus colonies. MRI has its advantages in showing soft-tissue and vascular invasion; it offers the possibility of differentiation between inflammatory tissue of sinus, mycetoma and neoplasm (22).

The diagnosis of an ophthalmologist was central retinal artery occlusion and effective action must be taken within the first 90 minutes (23). On this basis, it is advisable to consult an ophthalmologist in similar diseases. It is necessary that the disease is diagnosed and treated immediately. Facial nerve paralysis had led to right eye proptosis and numbness of lower jaw. It is suggested that in patients with uncontrolled diabetes and no bleeding after tooth extraction, probably due to the thin alveolar canal, the risk of developing sinus infection will increase. In this context, antibiotic prophylaxis is required. This article provides several clinical and diagnostic hints for effectively and efficiently treatment of patients without leaving any inability.
